# AI models predicting breast cancer distant metastasis using LightGBM with clinical blood markers and ultrasound maximum diameter

**DOI:** 10.1038/s41598-024-66658-x

**Published:** 2024-07-06

**Authors:** Yang Tan, Wen-hai Zhang, Zhen Huang, Qi-xing Tan, Yue-mei Zhang, Chang-yuan Wei, Zhen-Bo Feng

**Affiliations:** 1 Department of Pathology, The First Affiliated Hospital of Guangxi Medical University, No.6 Shuangyong Road, Nanning, 530000 Guangxi Zhuang Autonomous Region China; 2 Department of Breast Surgery, Guangxi Medical University Tumor Hospital, 71 Hedi Road, Nanning, 530021 Guangxi Zhuang Autonomous Region China

**Keywords:** Breast cancer, Cancer screening

## Abstract

Breast cancer metastasis significantly impacts women's health globally. This study aimed to construct predictive models using clinical blood markers and ultrasound data to predict distant metastasis in breast cancer patients, ensuring clinical applicability, cost-effectiveness, relative non-invasiveness, and accessibility of these models. Analysis was conducted on data from 416 patients across two centers, focusing on clinical blood markers (tumor markers, liver and kidney function indicators, blood lipid markers, cardiovascular biomarkers) and maximum lesion diameter from ultrasound. Feature reduction was performed using Spearman correlation and LASSO regression. Two models were built using LightGBM: a clinical model (using clinical blood markers) and a combined model (incorporating clinical blood markers and ultrasound features), validated in training, internal test, and external validation (test1) cohorts. Feature importance analysis was conducted for both models, followed by univariate and multivariate regression analyses of these features. The AUC values of the clinical model in the training, internal test, and external validation (test1) cohorts were 0.950, 0.795, and 0.883, respectively. The combined model showed AUC values of 0.955, 0.835, and 0.918 in the training, internal test, and external validation (test1) cohorts, respectively. Clinical utility curve analysis indicated the combined model's superior net benefit in identifying breast cancer with distant metastasis across all cohorts. This suggests the combined model's superior discriminatory ability and strong generalization performance. Creatine kinase isoenzyme (CK-MB), CEA, CA153, albumin, creatine kinase, and maximum lesion diameter from ultrasound played significant roles in model prediction. CA153, CK-MB, lipoprotein (a), and maximum lesion diameter from ultrasound positively correlated with breast cancer distant metastasis, while indirect bilirubin and magnesium ions showed negative correlations. This study successfully utilized clinical blood markers and ultrasound data to develop AI models for predicting distant metastasis in breast cancer. The combined model, incorporating clinical blood markers and ultrasound features, exhibited higher accuracy, suggesting its potential clinical utility in predicting and identifying breast cancer distant metastasis. These findings highlight the potential prospects of developing cost-effective and accessible predictive tools in clinical oncology.

## Introduction

Breast cancer stands as one of the most prevalent malignancies affecting women's health globally. By 2020, it had ranked as the most common cancer worldwide^1^, placing fourth in cancer-related mortality, with the highest rise in new fatal cases attributed to breast cancer^1^. In China alone, there are approximately 416,371 new cases and 117,174 related deaths annually, accounting for approximately 18.41% and 17.11% of global cases, respectively^2^. The majority of deaths stem from metastasis, with an estimated 20–30% of breast cancer patients experiencing this progression^3^.

The sites of distant metastasis in breast cancer closely correlate with post-metastatic survival, with bone, lungs, and liver being the most common locations^3,4^. Presently, clinical diagnosis of breast cancer distant metastasis heavily relies on imaging techniques. For instance, MRI, when using multi-sequence comprehensive imaging, offers morphological and functional information for bone metastasis without ionizing radiation exposure. Chest CT is recommended for detecting lung metastasis, and combining CT and MRI aids in diagnosing liver metastasis^5,6^.

However, conventional imaging methods exhibit limitations in distinguishing breast cancer distant metastasis. Challenges include difficulties in differentiating benign nodules in the lungs from lung metastases and identifying atypical vascular tumors in liver metastasis diagnosis^5^. Moreover, these diagnostic procedures can be expensive, posing a significant financial burden, particularly for breast cancer patients in developing countries, necessitating multiple examinations.

In response to these challenges, research has started exploring the use of artificial intelligence (AI) to assist in predicting breast cancer distant metastasis^7–12^. AI-driven predictive approaches hold promise for delivering faster, more accurate diagnoses while potentially reducing the need for expensive imaging tests, thereby alleviating patients’ economic burdens. Current AI research on breast cancer distant metastasis primarily focuses on assessing the risk of future (1, 3, or 5 years) metastasis^7–12^. If breast cancer patients could undergo an evaluation for distant metastasis before relatively expensive whole-body imaging or relatively invasive pathological examinations, it might help avoid unnecessary whole-body imaging tests.

This study boldly attempts AI-based diagnosis for breast cancer with distant metastasis. By integrating clinical blood markers and ultrasound data, a novel AI model for distinguishing breast cancer distant metastasis was established. The novelty lies in its independence from costly and occasionally inaccessible imaging examinations, instead utilizing relatively accessible clinical blood markers and cost-effective ultrasound data for predicting breast cancer distant metastasis. This approach not only enhances diagnostic affordability and accessibility but also introduces a new avenue for early detection of breast cancer distant metastasis. With technological advancements and deeper research, AI's application in predicting breast cancer distant metastasis could become a crucial future development in this field.

## Materials and methods

### Patients population

**Figure 1:**
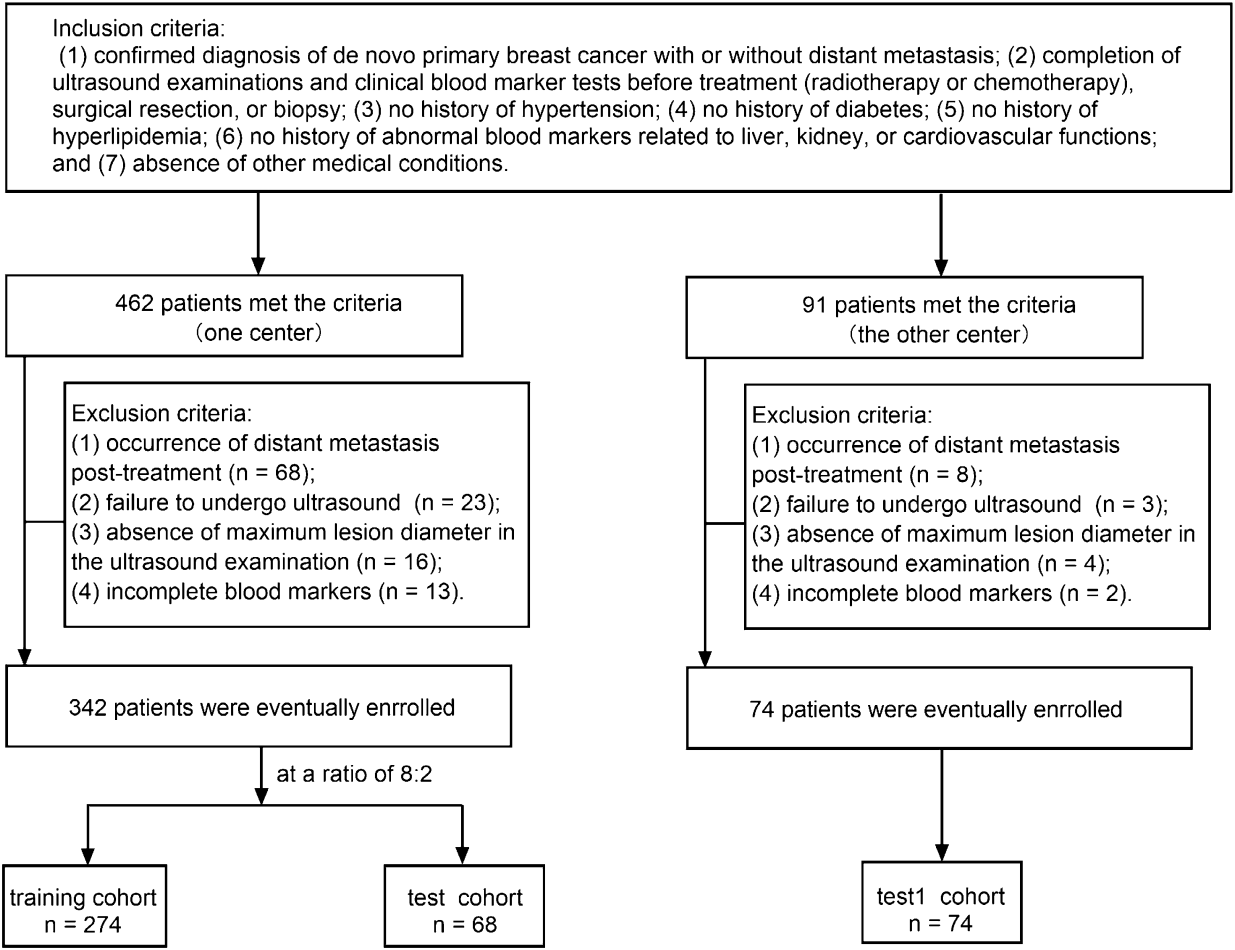
The flowchart for selecting the study patients.

**Figure 2:**
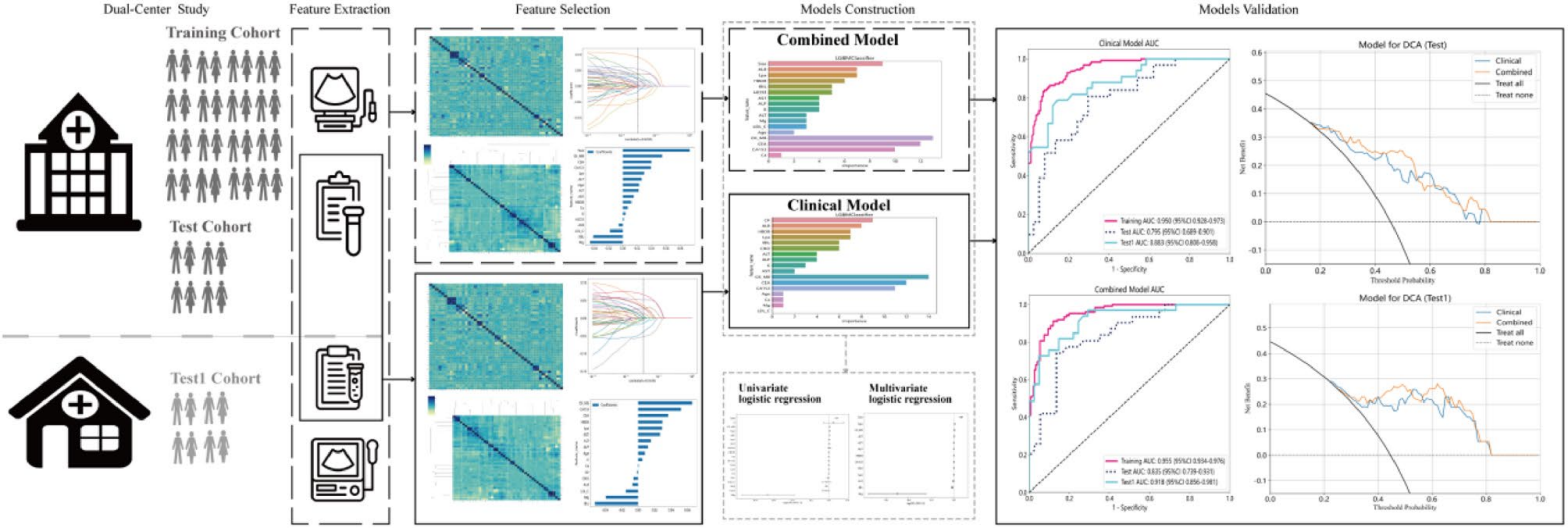
The workflow of clinical model and combined model in this study.

This retrospective study involved data from two centers, approved by the institutional review boards of both centers. All methods were performed in accordance with the relevant guidelines and regulations. Inclusion criteria were as follows: (1) confirmed diagnosis of de novo primary breast cancer with or without distant metastasis; (2) completion of ultrasound examinations and clinical blood marker tests before treatment (radiotherapy or chemotherapy), surgical resection, or biopsy; (3) no history of hypertension; (4) no history of diabetes; (5) no history of hyperlipidemia; (6) no history of abnormal blood markers related to liver, kidney, or cardiovascular functions; and (7) absence of other medical conditions. Exclusion criteria comprised: (1) occurrence of distant metastasis post-treatment (surgical resection or chemotherapy); (2) failure to undergo ultrasound due to unavoidable reasons (e.g., breast surface dressing); (3) absence of maximum lesion diameter in the ultrasound examination; and (4) lack of tumor markers (AFP, CEA, CA125, CA153, and CA199), liver function, kidney function, lipid profile, or cardiovascular function in the clinical blood markers. Breast cancer cases involved in this study were sourced from two research centers, with 342 cases from one center divided randomly in an 8:2 ratio into training (274 cases) and test (68 cases) sets, and 74 cases from the other center forming an external testing (test1) set. Given that breast cancer distant metastasis predominantly occurs in bones, lungs, and liver, the study included cases of bone, lung, and liver metastases among the breast cancer distant metastasis cases, as detailed in Table 1. The flowchart for selecting the study patients is shown in Fig. 1. The workflow of the models in this study is depicted in Fig. 2.

**Table 1:** Clinical blood markers, pathological, and ultrasound characteristics in the training, test, and test1 cohorts.

Characteristics	Training cohort (n = 274)			Test cohort (n = 68)			Test1 cohort (n = 74)			
	Non-distant metastasis (n = 150)	Distant metastasis (n = 124)	*P* value	Non-distant metastasis (n = 37)	Distant metastasis (n = 31)	*P* value	Non-distant metastasis (n = 41)	Distant metastasis (n = 33)		*P* value
Age (years), mean ± SD	48.47 ± 8.96	50.85 ± 10.94	0.049	45.89 ± 10.21	52.52 ± 12.59	0.019	50.63 ± 11.65		50.82 ± 9.53	0.942
Weight (kg), mean ± SD	58.38 ± 7.84	56.30 ± 8.40	0.036	58.99 ± 9.35	55.37 ± 8.45	0.102	57.55 ± 9.87		55.44 ± 7.60	0.317
Maximum tumor diameter by ultrasound (cm), mean ± SD	3.52 ± 1.44	5.04 ± 2.82	< 0.001	3.46 ± 1.97	4.69 ± 2.48	0.025	2.00 ± 0.82		4.39 ± 1.31	< 0.001
CEA (ng/ml)	2.73 ± 3.93	22.20 ± 79.45	0.003	3.22 ± 6.66	25.13 ± 46.77	0.006	3.19 ± 5.44		206.97 ± 834.57	0.122
AFP (ng/ml)	3.03 ± 2.08	15.22 ± 128.70	0.247	2.74 ± 1.09	2.61 ± 1.16	0.632	2.43 ± 0.96		3.48 ± 3.60	0.075
CA125 (U/ml)	29.50 ± 82.97	57.94 ± 154.19	0.053	37.76 ± 104.34	61.06 ± 178.39	0.505	18.85 ± 18.49		283.28 ± 945.41	0.077
CA153 (U/ml)	20.92 ± 56.20	90.15 ± 159.84	< 0.001	19.45 ± 20.73	80.70 ± 156.61	0.021	15.01 ± 10.38		243.32 ± 725.40	0.047
CA199 (U/ml)	15.67 ± 35.81	21.77 ± 36.56	0.166	19.84 ± 39.05	42.25 ± 89.81	0.175	10.99 ± 12.85		118.00 ± 439.24	0.123
TBIL (μ mol/L)	12.88 ± 4.60	11.19 ± 4.15	0.002	13.75 ± 7.85	11.45 ± 3.69	0.138	13.67 ± 4.43		23.22 ± 39.07	0.124
DBIL (μ mol/L)	3.99 ± 1.58	3.73 ± 1.48	0.173	4.24 ± 2.37	3.85 ± 1.38	0.412	3.37 ± 1.40		11.76 ± 28.69	0.065
IBIL (μ mol/L)	8.89 ± 3.26	7.29 ± 2.48	< 0.001	9.51 ± 5.55	7.60 ± 2.47	0.081	10.30 ± 3.48		11.46 ± 12.30	0.568
TP (g/L)	70.12 ± 8.34	69.15 ± 6.87	0.299	69.74 ± 6.10	69.80 ± 5.84	0.971	71.27 ± 6.10		71.40 ± 7.01	0.933
ALB (g/L)	40.92 ± 4.69	38.72 ± 5.18	< 0.001	40.86 ± 3.09	38.71 ± 3.36	0.008	40.13 ± 2.79		37.20 ± 4.85	0.002
GLO (g/L)	29.29 ± 4.31	29.98 ± 5.08	0.222	28.81 ± 4.67	31.09 ± 4.85	0.053	31.13 ± 5.37		34.20 ± 5.54	0.019
A_G (Ratio)	1.42 ± 0.21	1.32 ± 0.25	< 0.001	1.45 ± 0.21	1.28 ± 0.24	0.002	1.32 ± 0.26		1.11 ± 0.24	< 0.001
GGT (U/L)	22.24 ± 16.70	36.21 ± 43.78	< 0.001	18.85 ± 9.32	28.29 ± 20.35	0.014	19.34 ± 10.62		102.33 ± 248.82	0.036
TBA (μ mol/L)	6.56 ± 9.71	5.98 ± 8.50	0.605	3.33 ± 2.66	4.47 ± 4.24	0.179	7.41 ± 13.12		23.04 ± 45.90	0.041
PA (mg/L)	245.95 ± 53.55	231.07 ± 147.68	0.253	245.55 ± 43.40	237.55 ± 103.49	0.670	251.98 ± 37.22		209.78 ± 63.03	< 0.001
AST (U/L)	25.35 ± 10.58	34.80 ± 24.15	< 0.001	23.73 ± 6.69	32.65 ± 17.83	0.006	20.93 ± 7.62		95.91 ± 256.37	0.065
ALT (U/L)	16.85 ± 9.64	24.84 ± 25.54	< 0.001	16.43 ± 8.46	21.13 ± 14.53	0.102	13.54 ± 5.11		38.58 ± 70.70	0.027
AST_ALT (Ratio)	1.85 ± 1.63	1.75 ± 0.74	0.521	1.67 ± 0.61	1.73 ± 0.61	0.690	1.68 ± 0.72		2.14 ± 1.35	0.060
ALP (U/L)	66.17 ± 21.76	97.46 ± 88.77	< 0.001	58.70 ± 19.52	98.58 ± 51.63	< 0.001	62.00 ± 20.08		161.55 ± 257.89	0.016
CHE (U/L)	8979.22 ± 2168.44	8418.73 ± 2342.68	0.041	8716.92 ± 2206.69	8256.81 ± 2010.96	0.376	8740.63 ± 1614.03		8026.85 ± 2500.59	0.142
UREA (mmol/L)	7.28 ± 32.64	4.92 ± 2.41	0.423	4.39 ± 1.89	5.13 ± 1.62	0.088	4.47 ± 1.24		4.64 ± 1.42	0.575
CREA (μ mol/L)	60.61 ± 16.18	63.38 ± 23.61	0.253	61.41 ± 26.01	65.71 ± 19.77	0.452	60.78 ± 10.52		61.09 ± 16.50	0.922
UA (μ mol/L)	311.17 ± 82.31	311.82 ± 98.75	0.952	295.86 ± 95.90	300.34 ± 89.02	0.844	311.80 ± 71.65		314.15 ± 110.43	0.912
HCO3 (mmol/L)	25.60 ± 2.78	25.81 ± 2.29	0.509	25.92 ± 2.05	26.13 ± 2.92	0.729	25.43 ± 2.69		24.57 ± 2.76	0.182
Ccr (ml/min)	97.37 ± 23.12	90.62 ± 26.69	0.026	102.64 ± 26.66	90.21 ± 22.43	0.044	99.57 ± 17.73		96.58 ± 24.84	0.548
CYSC (mg/L)	1.49 ± 8.10	0.90 ± 0.30	0.424	0.82 ± 0.31	0.87 ± 0.22	0.437	0.80 ± 0.15		0.85 ± 0.26	0.232
K (mmol/L)	3.95 ± 0.36	4.01 ± 0.36	0.149	3.98 ± 0.31	3.80 ± 0.44	0.055	3.94 ± 0.34		4.03 ± 0.45	0.363
Na (mmol/L)	139.75 ± 11.06	139.68 ± 12.55	0.961	141.38 ± 2.70	141.06 ± 2.72	0.636	139.64 ± 2.00		139.03 ± 3.16	0.319
Cl (mmol/L)	102.05 ± 7.59	101.20 ± 9.46	0.410	102.57 ± 2.66	99.08 ± 18.38	0.257	105.84 ± 2.12		104.95 ± 3.39	0.171
Ca (mmol/L)	2.28 ± 0.21	18.53 ± 180.56	0.271	2.28 ± 0.14	2.32 ± 0.27	0.526	2.26 ± 0.09		2.32 ± 0.13	0.034
Mg (mmol/L)	1.02 ± 0.11	0.97 ± 0.13	< 0.001	1.03 ± 0.11	0.97 ± 0.16	0.060	0.90 ± 0.19		0.86 ± 0.08	0.225
PHO (mmol/L)	1.67 ± 7.10	1.12 ± 0.20	0.395	1.06 ± 0.12	1.13 ± 0.16	0.054	1.13 ± 0.13		1.14 ± 0.19	0.748
CK (U/L)	84.97 ± 50.30	75.65 ± 35.65	0.084	83.68 ± 35.03	81.16 ± 41.34	0.787	80.00 ± 56.13		81.42 ± 44.96	0.906
CK_MB (U/L)	11.15 ± 9.48	21.35 ± 23.72	< 0.001	10.22 ± 6.80	21.97 ± 41.75	0.096	12.95 ± 3.66		21.97 ± 15.47	< 0.001
LDH (U/L)	176.38 ± 47.17	263.37 ± 317.11	0.001	173.78 ± 39.25	201.16 ± 79.96	0.071	150.68 ± 28.97		289.76 ± 195.29	< 0.001
HBDB (U/L)	129.00 ± 28.83	223.72 ± 339.16	< 0.001	128.27 ± 39.03	153.71 ± 48.85	0.020	110.05 ± 21.17		214.61 ± 170.89	< 0.001
CHO (mmol/L)	5.28 ± 1.14	4.98 ± 0.99	0.025	5.01 ± 1.03	5.02 ± 0.71	0.950	5.13 ± 1.00		5.35 ± 1.37	0.440
TG (mmol/L)	1.36 ± 0.94	1.37 ± 0.88	0.881	1.49 ± 1.13	1.41 ± 0.77	0.736	1.30 ± 0.86		43.53 ± 241.00	0.265
HDL_C (mmol/L)	1.42 ± 0.37	1.34 ± 0.36	0.056	1.29 ± 0.39	1.30 ± 0.23	0.841	1.39 ± 0.26		1.26 ± 0.34	0.076
LDL_C (mmol/L)	3.19 ± 0.99	2.93 ± 0.85	0.022	2.95 ± 0.86	3.04 ± 0.66	0.649	3.08 ± 0.84		3.45 ± 1.34	0.152
APO_A1 (g/L)	1.35 ± 0.21	2.51 ± 13.54	0.295	1.30 ± 0.20	1.27 ± 0.15	0.472	1.34 ± 0.22		1.23 ± 0.28	0.044
APO_B (g/L)	0.99 ± 0.28	1.50 ± 6.39	0.330	1.06 ± 0.43	0.94 ± 0.22	0.161	0.86 ± 0.22		0.95 ± 0.30	0.142
A1_B1 (Ratio)	1.47 ± 0.44	1.53 ± 0.58	0.279	1.43 ± 0.38	1.42 ± 0.33	0.932	1.66 ± 0.48		1.37 ± 0.50	0.012
Lpa (mg/L)	243.74 ± 236.81	340.65 ± 302.95	0.003	220.92 ± 230.21	308.81 ± 339.66	0.210	265.44 ± 259.38		260.82 ± 221.40	0.935
Pathological diagnosis*, n (%)			0.688			0.875				0.279
Infiltrating duct carcinoma, NOS	143(95.33)	115(92.74)		36(97.30)	29(93.55)		41(100.00)		31(93.94)	
Infiltrating lobular carcinoma, NOS	3(2.00)	4(3.23)		1(2.70)	2(6.45)		0(0.00)		1(3.03)	
Invasive papillary carcinoma	0(0.00)	1(0.81)		0(0.00)	0(0.00)		0(0.00)		0(0.00)	
Invasive micropapillary carcinoma	2(1.33)	2(1.61)		0(0.00)	0(0.00)		0(0.00)		0(0.00)	
Mucinous adenocarcinoma	1(0.67)	2(1.61)		0(0.00)	0(0.00)		0(0.00)		1(3.03)	
Metaplastic carcinoma	1(0.67)	0(0.00)		0(0.00)	0(0.00)		0(0.00)		0(0.00)	
Lymph node metastasis*, n (%)			–			–				–
Absent	104(69.33)	10(8.06)		24(64.86)	0(0.00)		32(78.05)		3(9.09)	
Present	46(30.67)	114(91.94)		13(35.14)	31(100.00)		9(21.95)		30(90.91)	
Distant metastasis*, n (%)			–			–				–
Bone metastasis	–	65(52.42)		–	21(67.74)		–		17(51.52)	
Lung metastasis	–	35(28.23)		–	6(19.35)		–		8(24.24)	
Liver metastasis	–	24(19.35)		–	4(12.90)		–		8(24.24)	

SD, Standard deviation; AFP, Alpha-Fetoprotein; CEA, Carcinoembryonic Antigen; CA125, Carbohydrate Antigen125; CA153, Carbohydrate Antigen153; CA199, Carbohydrate Antigen 199; TBIL, Total bilirubin; DBIL, Direct bilirubin; IBIL, Indirect bilirubin; TP, Total protein; ALB, Albumin; GLO, globulin; A_G (Ratio), Albumin-globulin ratio; GGT, γ-glutamyl transferase; TBA, Total bile acids; PA, Pre-albumin; AST, Aspartate aminotransferase; ALT, Alanine aminotransferase; AST_ALT (Ratio), Aspartate aminotransferase to alanine aminotransferase ratio; ALP, Alkaline phosphatase; CHE, Cholinesterase; UREA, Urea; CREA, Creatinine; UA, Uric acid; HCO3, Blood bicarbonate concentration; Ccr, endogenous creatinine clearance rate; CYSC, Cysteine Protease Inhibitor C; K, Potassium ion; Na, Sodium ion; Cl, chloride ion; Ca, Ccalcium ion; Mg, Magnesium ion; PHO, Inorganic phosphate; CK, Creatine kinase; CK_MB, Creatine kinase isoenzyme; LDH, Lactate dehydrogenase; HBDB, α-hydroxybutyrate dehydrogenase; CHO, Total cholesterol; TG, Triglycerides; HDL_C, High-density lipoprotein cholesterol; LDL_C, Low-density lipoprotein cholesterol; APO_A1, Apolipoprotein A1; APO_B, Apolipoprotein B; A1_B (Ratio), Apolipoprotein A1 to apolipoprotein B ratio; Lpa, lipoprotein (a).

*Indicates that this characteristic does not need to be included in model construction.

### Feature extraction and selection

Features extracted from clinical blood markers included tumor markers (carcinoembryonic antigen, alpha-fetoprotein, CA125, CA153, and CA199), liver function indicators (total bilirubin, direct bilirubin, indirect bilirubin, total protein, albumin, globulin, albumin-globulin ratio, gamma-glutamyl transferase, prealbumin, aspartate transaminase (AST), alanine transaminase (ALT), AST/ALT ratio, alkaline phosphatase, cholinesterase, and total bile acid), kidney function indicators (urea, creatinine, uric acid, blood bicarbonate concentration, cystatin C, potassium ion, sodium ion, chloride ion, calcium ion, and inorganic phosphorus), lipid profile (total cholesterol, triglycerides, high-density lipoprotein cholesterol, low-density lipoprotein cholesterol, apolipoprotein A1, apolipoprotein B, A1/B ratio, and lipoprotein (a)), and cardiovascular function indicators (creatine kinase, creatine kinase isoenzyme (CK-MB), lactate dehydrogenase, and alpha-hydroxybutyrate dehydrogenase). Features from ultrasound data included the maximum diameter of breast cancer lesions.

All extracted features underwent the following procedures: initial standardization using z-score normalization (mean of 0, standard deviation of 1) to achieve a standard normal distribution of the data. Subsequently, statistical analysis employing Spearman's rank correlation coefficient measured the correlation between pairs of variables. When the Spearman correlation coefficient between features was > 0.9, one of the correlated features was retained. Then, we used the Least Absolute Shrinkage and Selection Operator (LASSO) regression model for feature selection. LASSO is a regression method that introduces an L1 regularization term, which shrinks some of the regression coefficients to zero, thereby achieving feature selection. During the feature selection process, we used Mean Squared Error (MSE) to determine the optimal regularization parameter (λ) for the LASSO model. Specifically, we calculated the MSE for different λ values through cross-validation and chose the λ value that minimized the MSE as the optimal parameter. The purpose of using MSE is to find a feature subset that effectively reduces model complexity while maintaining good predictive performance. In summary, using L1 regularized LASSO regression for feature dimensionality reduction eliminates highly correlated features, generating a sparse model where only a few features significantly contribute to the predictive outcomes, thereby enhancing the model's interpretability and generalizability.

### Development and validation of models

This study employed the LightGBM machine learning algorithm to construct models for breast cancer with distant metastasis, utilizing the dimensionality-reduced features. The learning rate in the LightGBM model significantly impacts the convergence speed and performance of the model. Through cross-validation, we selected an appropriate learning rate to balance the learning speed and accuracy of the model during training. The number of trees and the depth of the trees in the LightGBM model directly affect the complexity and fitting ability of the model. We determined the optimal number of trees and tree depth through grid search methods to avoid overfitting or underfitting. The selected features from clinical blood markers were used to build the clinical model, while a combination of clinical blood markers and ultrasound features, post-dimensionality reduction, constituted the combined model. Model construction was based on fivefold cross-validation of the training set. Following model construction, validation was performed on internal (test) and external test (test1) sets, evaluating performance using metrics such as area under the curve (AUC), accuracy, sensitivity, specificity, positive predictive value, and negative predictive value. Subsequently, clinical decision curve analysis (DCA) was conducted, depicting the net benefit at different probability thresholds in training and internal–external validation sets to assess the clinical efficiency of the model.

### Statistical analysis

Clinical baseline characteristics underwent t-tests, chi-square tests, or Fisher’s exact tests using SPSS software (version 25.0, IBM). The t-test was employed for continuous variables with homogeneity of variance, presented as x ± s, while chi-square tests or Fisher's exact tests were used for categorical variables presented as ratios. A two-tailed *p* value < 0.05 indicated statistical significance. Spearman rank correlation tests, heatmap plotting, z-score normalization, univariate regression analysis, multivariate regression analysis, output of feature importance in LightGBM models, and LASSO regression analysis were performed using Python software (version 3.7.17; http://www.python.org). Additionally, receiver operating characteristic (ROC) curve and clinical decision curve plotting were conducted. The DeLong test was implemented using R (version 4.3.3).

### Ethical approval and consent to participate

This study has obtained ethical approval from the Medical Ethics Committee of the First Affiliated Hospital of Guangxi Medical University (Reference Number: 2023-E749-01) and the Medical Ethics Committee of Guangxi Medical University Tumor Hospital (Reference Number: KY2023868). Due to the retrospective nature of the study, the requirement for informed consent has been waived by the Medical Ethics Committee of the First Affiliated Hospital of Guangxi Medical University and the Medical Ethics Committee of Guangxi Medical University Tumor Hospital.

## Results

### Patient characteristics

This study encompassed data from two research centers involving a total of 416 female breast cancer cases. Among these, one center comprised 274 cases in the training cohort, 68 cases in the tese cohort, and the other center included 74 cases in the test1 cohort. Statistical differences existed across the three cohorts in blood markers including CA153, albumin, albumin-globulin ratio, gamma-glutamyl transferase, alkaline phosphatase, and alpha-hydroxybutyrate dehydrogenase, as well as the maximum diameter of breast cancer lesions obtained from ultrasound examinations. A summary of patient ultrasound and clinical blood marker features is provided in Table 1.

### Feature selection

**Figure 3:**
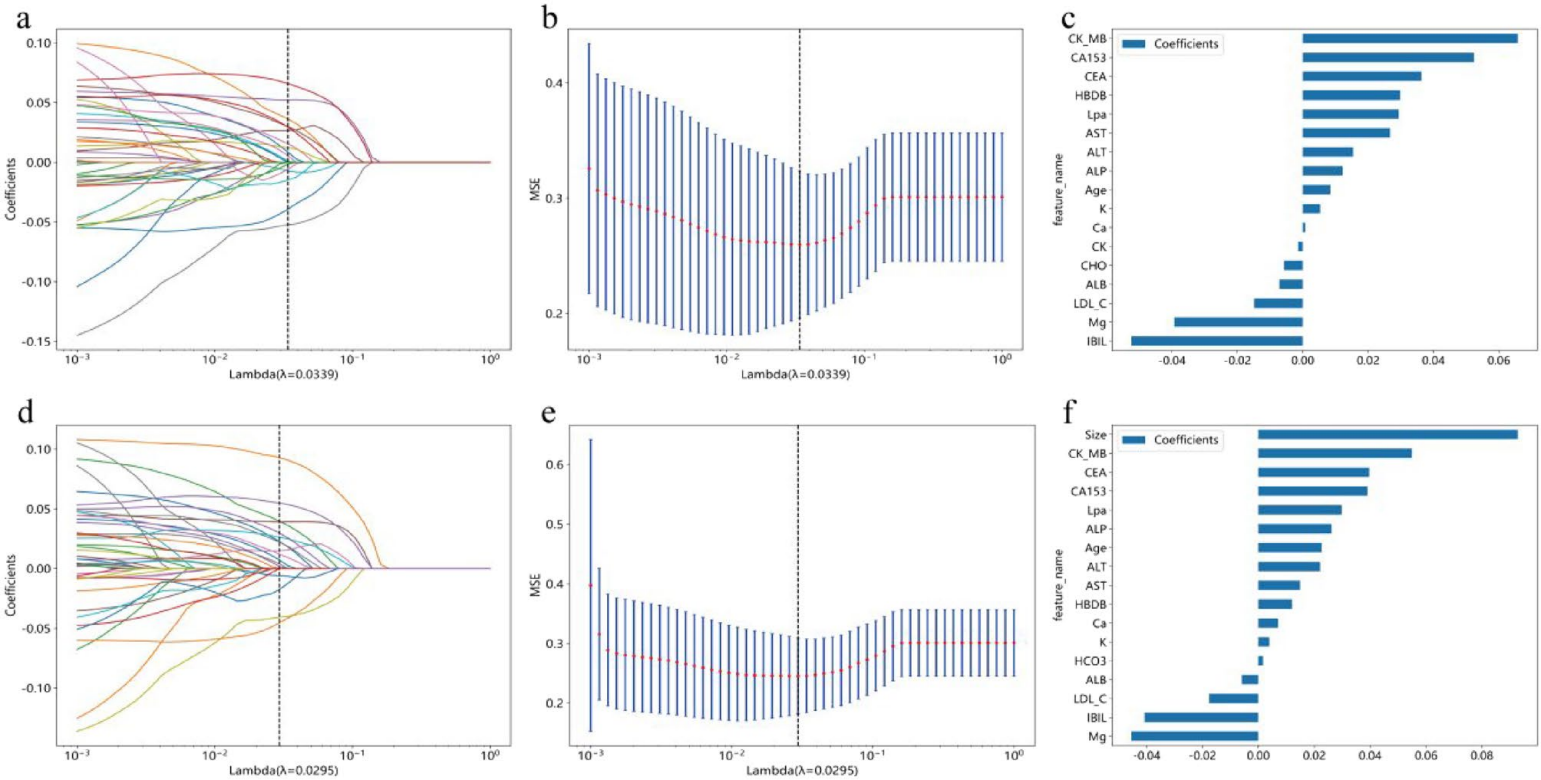
Illustrates the process of feature selection using the least absolute shrinkage and selection operator (LASSO) regression model. (**a**–**c**) Feature selection for clinical model; (**d**–**f**) Feature selection for combined model. (**a**, **d**) LASSO coefficients for different λ values, where vertical dashed lines indicate the number of features corresponding to the optimal λ value (clinical, 17; combined, 17). (**b**, **e**) Optimal λ values are chosen based on tenfold cross-validation and minimum mean squared error (MSE), represented by vertical dashed lines. After feature selection using least absolute shrinkage and selection operator regression, the nonzero coefficient features are as follows: (**c**) clinical features; (**f**) combined features.

The feature data underwent normalization, followed by the retention of one feature among those with a Spearman correlation coefficient > 0.9. A heatmap illustrating the correlation analysis of features is presented in Supplementary Fig. 1. Clinical blood marker features were utilized to construct the clinical model predicting breast cancer distant metastasis, while the combination of clinical blood markers and ultrasound features was used for the combined model. Dimensionality reduction was achieved by eliminating features with zero coefficients through LASSO regression. The optimal λ value was determined for fitting the Lasso regression model (Fig. 3a,d) based on the minimum Mean Squared Error (MSE) (Fig. 3b,e). Following feature dimensionality reduction, a final selection of 17 features was made in both instances (Fig. 3c,f).

### Construction and validation of clinical and combined models

**Figure 4:**
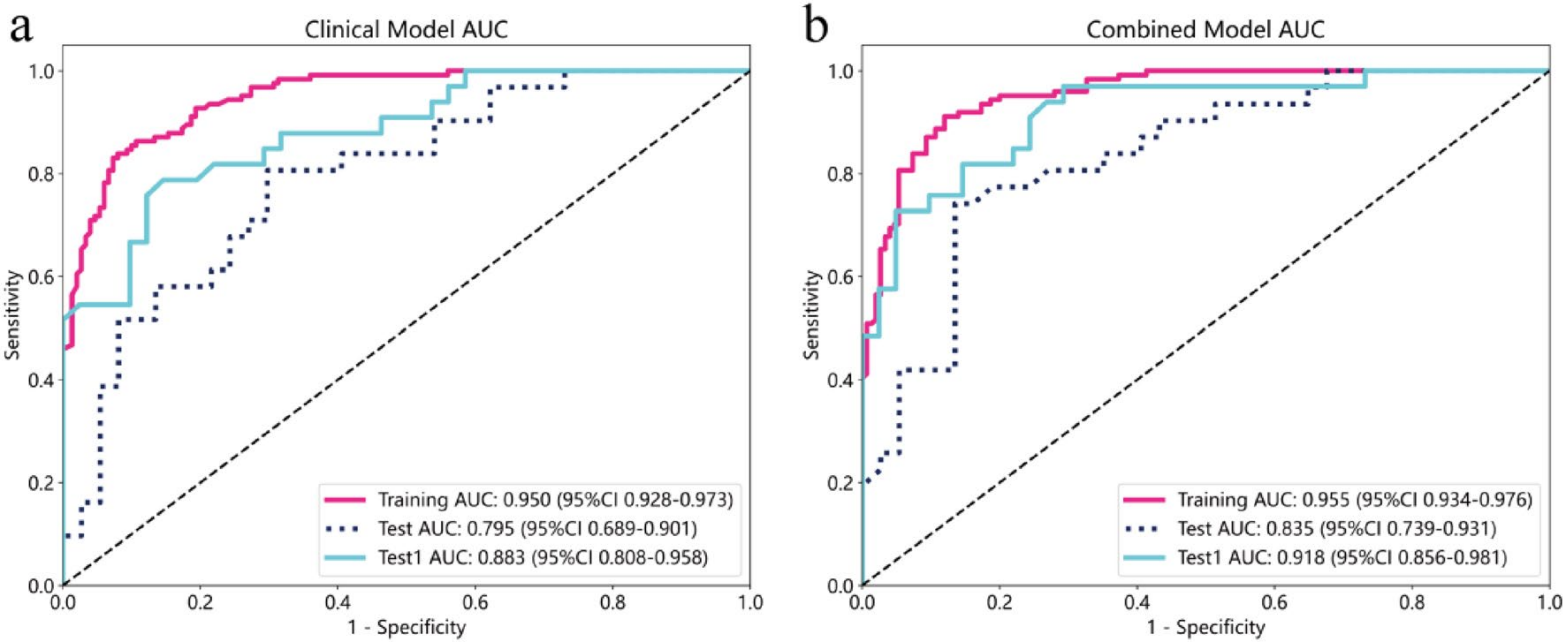
Evaluation of Receiver Operating Characteristic curves for the clinical (**a**) and combined (**b**) models constructed in both the training, test and test1 cohorts were presented.

**Figure 5:**
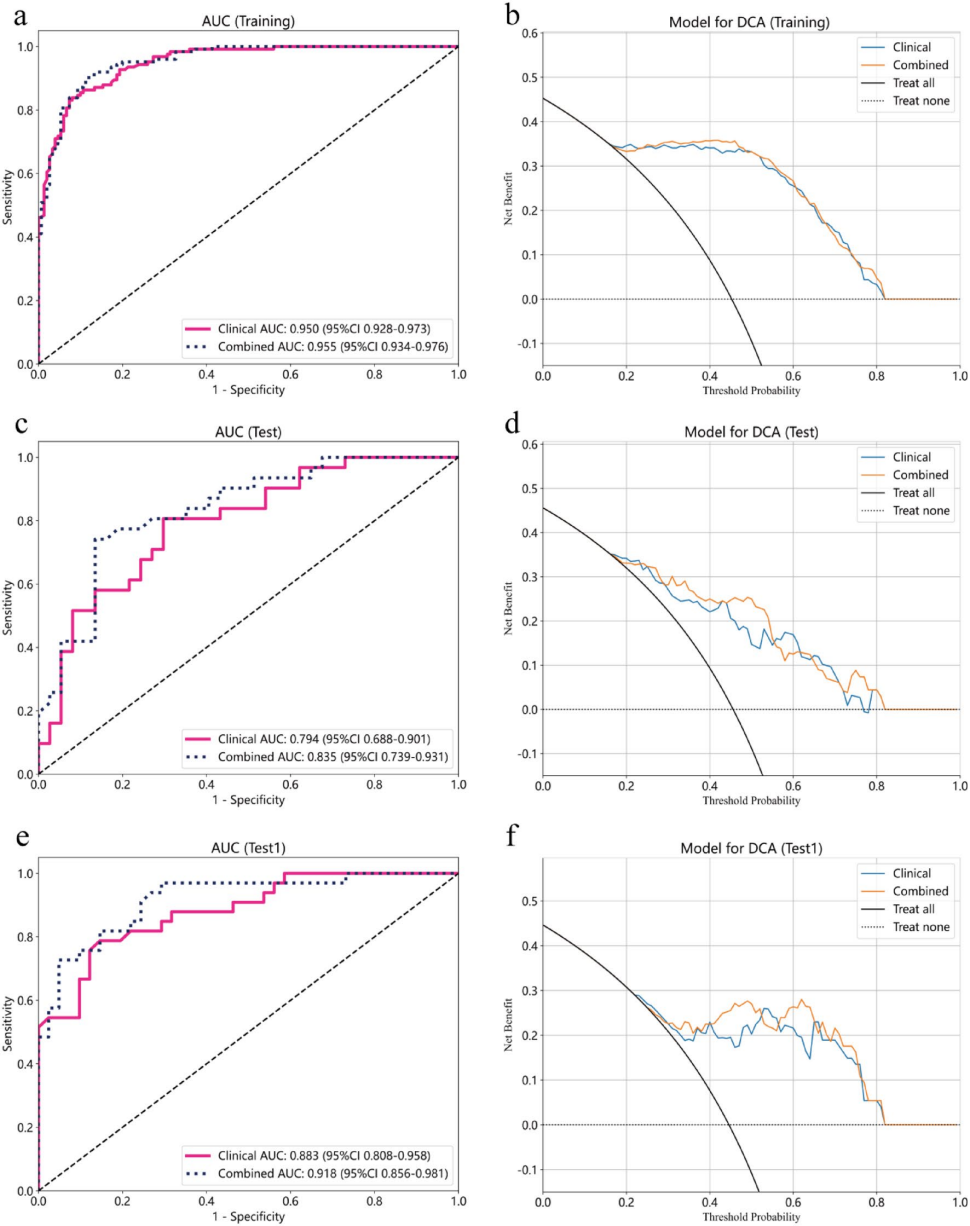
Receiver operating characteristic (ROC) curve evaluation and clinical decision curves analysis (DCA) for the clinical and combined models constructed in the training (**a**, ROC; **b**, DCA), test (**c**, ROC; **d**, DCA), and test1 (**e**, ROC; **f**, DCA) cohorts were demonstrated.

The LightGBM machine learning algorithm was employed to construct Clinical and combined models using the aforementioned selected features. The ROC curves for the clinical and combined models are displayed in Fig. 4a,b. The AUC values for the training, test, and test1 cohorts of the clinical model were 0.950 (95% CI 0.928–0.973), 0.795 (95% CI 0.689–0.901), and 0.883 (95% CI 0.808–0.958) respectively. For the combined model, the AUC values were 0.955 (95% CI 0.934–0.976), 0.835 (95% CI 0.739–0.931), and 0.918 (95% CI 0.856–0.981) for the training, test, and test1 cohorts respectively. Additional performance parameters are presented in Table 2. Notably, across the training, test, and test1 cohorts, the AUC values of the combined model were higher than those of the clinical model (Fig. 5a,c,e). DeLong tests were performed to assess differences in AUC values between the combined and clinical models specifically for the test and test1 cohorts. The DeLong test results indicated that the P-values comparing the AUC values of the combined model with the clinical model were both greater than 0.05 (Test cohort: *p* value 0.103; Test1 cohort: *p* value 0.245). Furthermore, DCA curves of these two models across the training, test, and test1 cohorts are depicted in Fig. 5b,d,f. The results indicate that the combined model exhibited the most significant net benefit in identifying breast cancer with distant metastasis across all three cohorts.

**Table 2:** Performance of models for predicting discrimination between breast cancer with distant metastasis and breast cancer without distant metastasis in training, test, and test1 cohorts.

Model	Cohort	AUC (95% CI)	Accuracy	Sensitivity	Specificity	PPV	NPV	Precision	Recall	F1	Threshold
Clinical	Training	0.950 (0.928–0.973)	0.880	0.831	0.920	0.896	0.868	0.896	0.831	0.862	0.499
	Test	0.794 (0.688–0.901)	0.735	0.774	0.703	0.686	0.788	0.686	0.774	0.727	0.442
	Test1	0.883 (0.808–0.958)	0.824	0.758	0.878	0.833	0.818	0.833	0.758	0.794	0.535
Combined	Training	0.955 (0.934–0.976)	0.891	0.903	0.880	0.862	0.917	0.862	0.903	0.882	0.460
	Test	0.835 (0.739–0.931)	0.794	0.710	0.865	0.815	0.780	0.815	0.710	0.759	0.508
	Test1	0.918 (0.856–0.981)	0.838	0.697	0.951	0.920	0.796	0.920	0.697	0.793	0.630

AUC, Area under the curve; PPV, Positive predictive value; NPV, Negative predictive value; F1, F1 Score.

### Analysis of model feature importance

**Figure 6:**
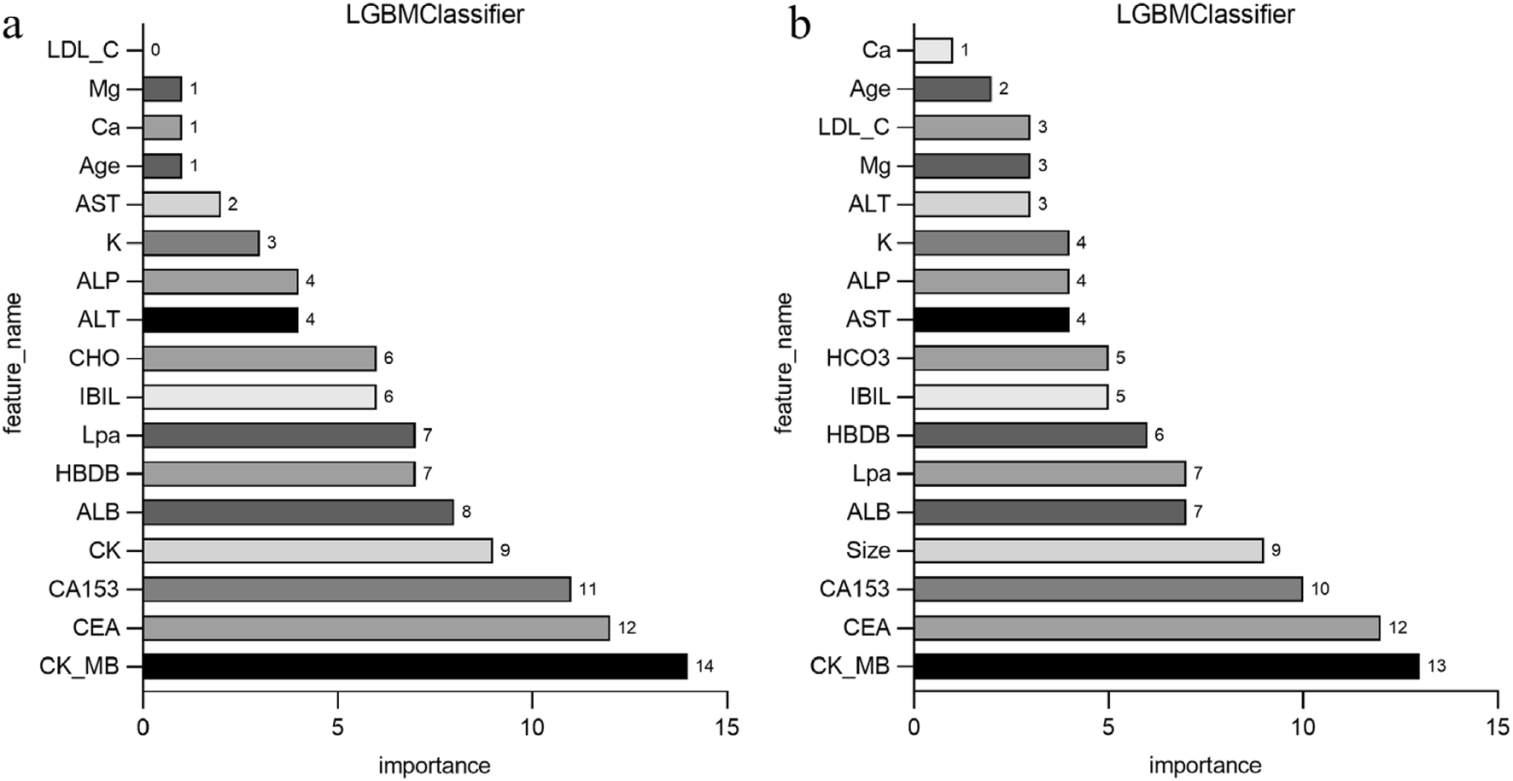
Feature importance analysis of clinical model (**a**) and combined model (**b**) built on the LightGBM algorithm. The color differences are used only for ease of identification and have no other meaning. The features shown are arranged in order of importance, and their degrees of importance are indicated numerically.

To identify the crucial clinical blood markers and ultrasound data features contributing significantly to the clinical and combined models' predictions of distant metastasis, feature importance analysis was conducted, as shown in Fig. 6a,b. The top 5 features from both the clinical and combined models were integrated, comprising CK-MB, CEA, CA153, albumin, creatine kinase, and the maximum diameter of lesions detected by ultrasound. Subsequently, univariate and multivariate regression analyses were performed on the involved features, displaying OR and *p* values in Table 3. Blood markers including CA153, indirect bilirubin, magnesium ion, CK-MB, lipoprotein (a), and the maximum diameter of lesions from ultrasound showed *p* values < 0.05 in both univariate and multivariate regression analyses, suggesting their potential association with breast cancer and metastasis. Among these, CA153, CK-MB, lipoprotein (a), and the maximum diameter of lesions from ultrasound exhibited positive correlations, while indirect bilirubin and magnesium ion showed negative correlations.

**Table 3:** Univariate and multivariate logistic regression analysis of variables (features) involved in models’ construction associated with breast cancer with distant metastasis.

Variables	Univariate analysis		Multivariate analysis	
	OR (95% CI)	*P* value	OR (95% CI)	*P* value
Age (years)	1.006 (1.002–1.010)	0.009	1.006 (1.003–1.010)	0.003
Maximum tumor diameter by ultrasound (cm)	1.085 (1.067–1.104)	< 0.001	1.067 (1.049–1.084)	< 0.001
CEA (ng/ml)	1.000 (1.000–1.000)	0.028	1.000 (1.000–1.000)	0.096
CA153 (U/ml)	1.000 (1.000–1.001)	< 0.001	1.000 (1.000–1.000)	0.021
IBIL (μ mol/L)	0.988 (0.979–0.996)	0.013	0.984 (0.974–0.994)	0.007
ALB (g/L)	0.974 (0.966–0.982	< 0.001	0.995 (0.987–1.004)	0.364
AST (U/L)	1.001 (1.000–1.001)	0.005	1.000 (0.999–1.001)	0.822
ALT (U/L)	1.004 (1.002–1.005)	< 0.001	1.002 (1.000–1.005)	0.147
ALP (U/L)	1.001 (1.001–1.002)	< 0.001	1.000 (1.000–1.001)	0.116
HCO3 (mmol/L)	1.001 (0.985–1.016)	0.932		
K (mmol/L)	1.050 (0.942–1.171)	0.459		
Ca (mmol/L)	1.000 (1.000–1.001)	0.270		
Mg (mmol/L)	0.518 (0.389–0.689)	< 0.001	0.608 (0.470–0.784)	0.001
CK (U/L)	0.999 (0.998–1.000)	0.152		
CK_MB (U/L)	1.007 (1.005–1.009)	< 0.001	1.003 (1.001–1.005)	0.018
HBDB (U/L)	1.001 (1.000–1.001)	< 0.001	1.000 (1.000–1.000)	0.274
CHO (mmol/L)	0.967 (0.931–1.004)	0.144		
LDL_C (mmol/L)	0.975 (0.934–1.017)	0.320		
Lpa (mg/L)	1.000 (1.000–1.000)	0.003	1.000 (1.000–1.000)	0.031

For detailed explanations of the abbreviations for the variables, please refer to Table 1.

## Discussion

This study utilized the LightGBM algorithm to construct clinical and combined models based on features derived from relatively easily accessible clinical blood markers and cost-effective routine ultrasonography, aiming to identify de novo breast cancer with distant metastasis. Both internal and external testing sets demonstrated superior performance. Additionally, the combined model exhibited greater net benefit in distinguishing breast cancer with distant metastasis, showcasing higher predictive efficiency and robustness. Our predictive models effectively discerned breast cancer patients with distant metastasis from those without, providing clinicians with additional suspicion evidence and potentially enabling more effective triage management in breast cancer diagnosis and treatment.

Breast cancer stands as the most common malignancy among women globally. Among these patients, distant metastasis represents a common form of recurrence and a lifelong risk they might encounter^13^. Notably, distant metastasis is a significant factor contributing to diminished quality of life and, in some cases, mortality among breast cancer patients^13,14^. Regarding the diagnosis of metastatic breast cancer, the European society for medical oncology clinical practice guidelines stipulate the necessity for confirmation through imaging studies such as MRI, CT scans, or functional imaging like positron emission tomography-computed tomography, dynamic contrast-enhanced magnetic resonance imaging, or magnetic resonance diffusion-weighted imaging when clinical suspicion exists^15^. The decision for breast cancer patients to undergo a series of imaging studies solely depends on clinicians' suspicion, often requiring expensive functional imaging studies even when results are inconclusive. The strength of our model lies in its ability to accurately identify patients who may have distant metastasis among those without it, providing clinicians with more clues and suspicion evidence.

This models' significance lies in providing a more effective method to identify patients potentially suffering from breast cancer with distant metastasis. It integrates clinical blood markers and ultrasound data, which are relatively easy to obtain and cost-effective. Through the analysis of these features, the model generates reliable predictions, guiding physicians to pay earlier attention to patient cohorts that might require further examination and monitoring. Specifically, the application of these models in clinical practice implies the ability to conduct more precise screening and diagnosis of breast cancer patients. Physicians can utilize these models to conduct further targeted examinations and assessments, especially for patients at risk of distant metastasis. This early identification and intervention can aid physicians in devising more personalized treatment plans, thereby improving patients' survival rates and quality of life.

Regarding the performance difference observed between the external validation set (test1 cohort) and the internal test set (test cohort), we carefully evaluated several factors that may have contributed to this disparity. Despite both centers adhering to similar inclusion and exclusion criteria, subtle differences in patient demographics, clinical practices, or data collection methods between centers could potentially influence model performance. Notably, the test1 cohort demonstrated superior results on multiple performance metrics compared to the test cohort, particularly in terms of higher AUC values for both the clinical and combined models (Fig. 4). This outcome underscores the robustness and generalizability of our developed models when applied to a completely independent dataset, validating their predictive capability across different patient populations and clinical settings.

We primarily assessed the model performance using AUC values and clinical decision curves. Despite the combined model achieving higher AUC values than the clinical model, it did not pass the DeLong test. However, clinical decision curves demonstrated that the combined model yielded greater net benefit than the clinical model in both the test and test1 cohorts. Therefore, we consider the performance of the combined model to be superior to that of the clinical model. The superiority of the integrated model lies not only in its superior predictive performance and clinical utility compared to clinical models, but more importantly in how it integrates multiple data sources to enhance decision support. Firstly, the integrated model combines clinical blood biomarkers and ultrasound features, representing hematological and imaging information respectively. This integration of diverse data allows the model to comprehensively consider patients' physiological, biochemical, and morphological characteristics, thereby enhancing the comprehensive and accurate prediction and diagnosis of distant metastasis in breast cancer. Secondly, relying solely on clinical blood biomarkers or a single imaging examination may lead to insufficient information or misjudgments. In contrast, the integrated model integrates multiple information sources to provide a more comprehensive assessment, reducing the risk of misdiagnosis and thereby increasing the precision and confidence of clinical decisions. Additionally, the development of the integrated model not only signifies scientific advancement but also opens new prospects for future clinical practice.

In addition to constructing artificial intelligence LightGBM models capable of identifying breast cancer with distant metastasis, this study conducted an analysis of the models' feature importance. Among the features involved in the models' predictions, CK-MB exhibited the most significant importance. CK-MB, a creatine kinase isoenzyme composed of M and B subunits, primarily exists in cardiac and skeletal muscles^16^. Chang et al. found significantly higher CK-MB-to-total-CK ratios in late-stage malignant tumors compared to early-stage ones^17^, suggesting an association between CK-MB and late-stage cancer. Li et al.’s^16^ research indicated elevated serum CK-MB activity in various cancers, including breast cancer, with significantly higher serum CK-MB activity in metastatic tumor patients. Regarding the origin of elevated CK-MB in malignant tumors, Lee et al. detected a higher proportion of CK-MB in tumor tissues of lung cancer patients, hypothesizing that increased plasma CK-MB originates from tumor tissues rather than cardiac or skeletal muscles^18^. In this study, CK-MB, as one of the important features of the model, played a significant role in the model’s predictions. In univariate and multivariate regression analyses, CK-MB emerged as an independent risk factor for breast cancer with distant metastasis, positively correlated with distant metastasis. Further research and exploration are required to understand why CK-MB elevation manifests in breast cancer with distant metastasis and its source, whether from tumors or other factors.

Regarding other features of the model, CA153, a common tumor marker, demonstrated the ability to predict breast cancer with distant metastasis^19^. In this study's model construction, CA153 was also one of the important features. Concerning magnesium ion concentration and breast cancer distant metastasis, Karki et al.^20,21^ detected lower magnesium ion concentrations in breast cancer distant metastasis. This study's findings align substantially with that, showing a negative correlation between magnesium ion concentration and breast cancer distant metastasis. Presently, studies regarding indirect bilirubin and its relation to prognosis exist for colorectal, ovarian, and lung cancers^22–24^. Liu et al.^25^ reported a significant association between hyperlipidemia and breast cancer distant metastasis. However, there are no reported connections between indirect bilirubin, lipoprotein (a), and breast cancer distant metastasis. This study, for the first time, attempts to incorporate these as features in predicting breast cancer distant metastasis using artificial intelligence models. The relationship between indirect bilirubin, lipoprotein (a), and breast cancer distant metastasis warrants further research and exploration.

This study also has some limitations. Firstly, the metastatic breast cancer cases included in this study were de novo breast cancer with distant metastasis, encompassing common bone, liver, and lung metastases. Compared to de novo metastatic breast cancer, the prognosis of post-treatment metastatic breast cancer is poorer, possibly due to the tumor molecular reselection after treatment, leading to more aggressive biology^26^. The included metastatic breast cancer in this study does not cover all types of distant metastases, such as brain metastasis and post-treatment breast cancer distant metastasis. Secondly, although our study data came from two centers, both belong to the same region. The source of the dataset lacks diversity, necessitating further verification across multiple centers, even internationally. Additionally, during the diagnostic process, the reporting habits and personal experience of ultrasound physicians varied significantly, leading to considerable heterogeneity in the ultrasound results. In our collected data, the maximum diameter of the lesion was mentioned in the vast majority of reports. To ensure data accuracy and reliability, we only included the maximum diameter in the ultrasound features. In future prospective studies, we will address these issues. For instance, we will ensure that all relevant ultrasound features (such as calcification and borders) are consistently mentioned in the reports, regardless of their presence or absence. To facilitate future data collection, we may use standardized forms for ultrasound physicians to complete. Finally, in different medical institutions or with different equipment, the model's performance might vary. This study's model might require more validation datasets to ensure its generalizability and robustness in various clinical settings.

## Conclusions

In summary, this study successfully developed and validated artificial intelligence clinical models and combined models using LightGBM machine learning algorithms based on clinical blood markers and ultrasound data to predict distant metastasis in breast cancer patients. Particularly, the combined model integrating clinical blood markers and ultrasound features exhibited high accuracy in predicting and identifying breast cancer distant metastasis, demonstrating potential clinical application value. These significant findings highlight the potential of developing economically efficient and easily obtainable predictive tools in clinical oncology. They are poised to elevate the level of clinical decision-making and prognosis assessment, potentially reducing the need for expensive or invasive imaging techniques. The research underscores the prospects of utilizing readily available clinical blood markers and cost-effective ultrasound data to develop predictive tools, holding critical significance for the advancement of clinical oncology, potentially offering patients more convenient and efficient healthcare.

### Supplementary Information

Supplementary Information 1.

Supplementary Information 2.

## Data Availability

All datasets generated for this study are included in the article/Supplementary Material.
