# Supplementary material for: AI models predicting breast cancer distant metastasis using LightGBM with clinical blood markers and ultrasound maximum diameter
Source: Sci Rep. 2024 Jul 6;14:15561. doi: 10.1038/s41598-024-66658-x (PMC11226620; doi:10.1038/s41598-024-66658-x)
Supplement: Supplementary file 1 — Supplementary Information 1. [file 41598_2024_66658_MOESM1_ESM.docx]

Supplementary Figure 1 The correlation analysis of features in the form of a heatmap, illustrating pairwise correlations between features. The areas where features intersect show correlation coefficients ranging from 0 to 1.
