# Supplementary material for: AI models predicting breast cancer distant metastasis using LightGBM with clinical blood markers and ultrasound maximum diameter
Source: Sci Rep. 2024 Jul 6;14:15561. doi: 10.1038/s41598-024-66658-x (PMC11226620; doi:10.1038/s41598-024-66658-x)
Supplement: Supplementary file 2 — Supplementary Information 2. [file 41598_2024_66658_MOESM2_ESM.pdf]

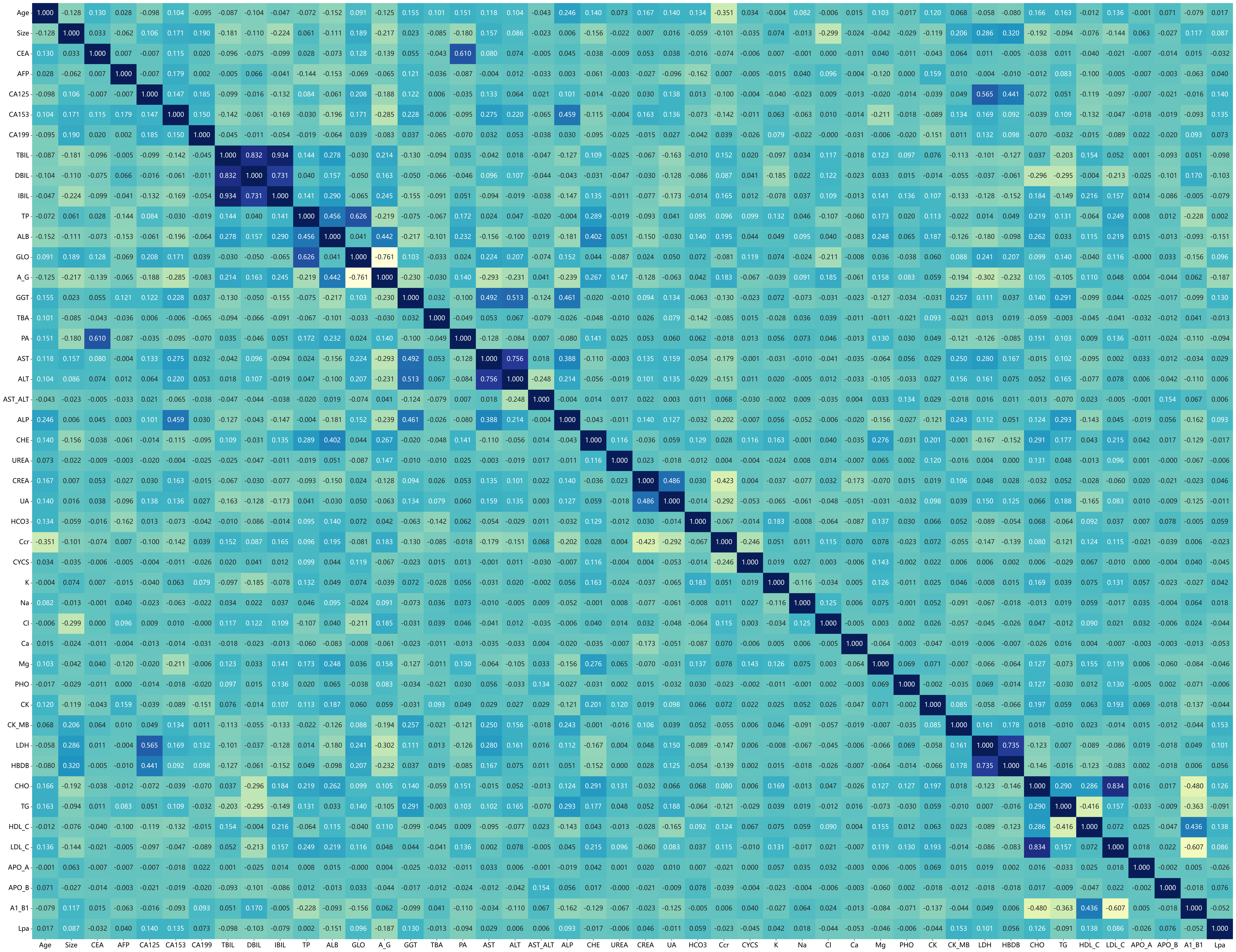
Age
Size
CEA
AFP
CA125
CA153
CA199
TBIL
DBIL
IBIL
TP
ALB
GLO
A_G
GGT
TBA
PA
AST
ALT
AST_ALT
ALP
CHE
UREA
CREA
UA
HCO3
Ccr
CYCS
K
Na
Cl
Ca
Mg
PHO
CK
CK_MB
LDH
HBDB
CHO
TG
HDL_C
LDL_C
APO_A
APO_B
A1_B1
Lpa
Age
Size
CEA
AFP
CA125
CA153
CA199
TBIL
DBIL
IBIL
TP
ALB
GLO
A_G
GGT
TBA
PA
AST
ALT
AST_ALT
ALP
CHE
UREA
CREA
UA
HCO3
Ccr
CYCS
K
Na
Cl
Ca
Mg
PHO
CK
CK_MB
LDH
HBDB
CHO
TG
HDL_C
LDL_C
APO_A
APO_B
A1_B1
Lpa
